# Calcium Sensor SlCBL4 Associates with SlCIPK24 Protein Kinase and Mediates Salt Tolerance in *Solanum lycopersicum*

**DOI:** 10.3390/plants10102173

**Published:** 2021-10-14

**Authors:** Joo Hyuk Cho, Sung-Chur Sim, Kyung-Nam Kim

**Affiliations:** 1 Department of Molecular Biology, Sejong University, Neungdong-ro 209, Gwangjin-gu, Seoul 05006, Korea; wngur1230@naver.com; 2 Department of Bioresource Engineering, Sejong University, Neungdong-ro 209, Gwangjin-gu, Seoul 05006, Korea; sungchur@sejong.ac.kr

**Keywords:** *Solanum lycopersicum*, calcium signaling, CBL4, CIPK24, salt tolerance

## Abstract

Soil salinity is one of the major environmental stresses that restrict the growth and development of tomato (*Solanum lycopersicum* L.) worldwide. In Arabidopsis, the calcium signaling pathway mediated by calcineurin B-like protein 4 (CBL4) and CBL-interacting protein kinase 24 (CIPK24) plays a critical role in salt stress response. In this study, we identified and isolated two tomato genes similar to the Arabidopsis genes, designated as SlCBL4 and SlCIPK24, respectively. Bimolecular fluorescence complementation (BiFC) and pull-down assays indicated that SlCBL4 can physically interact with SlCIPK24 at the plasma membrane of plant cells in a Ca2+-dependent manner. Overexpression of SlCBL4 or superactive SlCIPK24 mutant (SlCIPK24M) conferred salt tolerance to transgenic tomato (cv. Moneymaker) plants. In particular, the SlCIPK24M-overexpression lines displayed dramatically enhanced tolerance to high salinity. It is notable that the transgenic plants retained higher contents of Na+ and K+ in the roots compared to the wild-type tomato under salt stress. Taken together, our findings clearly suggest that SlCBL4 and SlCIPK24 are functional orthologs of the Arabidopsis counterpart genes, which can be used or engineered to produce salt-tolerant tomato plants.

## 1. Introduction

Tomato (*Solanum lycopersicum* L.) is one of the most economically important vegetable crops in the world, and yet its growth and development can be severely damaged by high salt concentration in soil [1]. In fact, soil salinity is a harmful environmental factor that significantly reduces the productivity of tomato and other vegetable crops around the world [2,3]. Therefore, a great deal of research has been conducted so far to elucidate the molecular mechanisms underlying the salt response of plants [4]. Such knowledge can ultimately be used to produce salt-tolerant vegetable plants and therefore improve their yield under salt stress [5].

Saline soil exerts its deleterious effect on plants in two distinct aspects, osmotic stress and ionic toxicity. At first, the osmotic event makes it difficult for the roots to absorb water and mineral nutrients from the soil due to the reduced soil water potential by dissolved salt ions. Thus, the plants are exposed to water deficit that reduces carbon assimilation and yield [6]. Following the osmotic stress, the ionic toxicity occurs to impose a detrimental effect by accumulating sodium ions (Na^+^) to a critical level in the leaves, which induces ionic imbalance in the cytosol of plant cells and leads to necrosis and early leaf senescence [2]. Na^+^ appears to lower the intracellular concentration of potassium ions (K^+^) in two ways, severely affecting K^+^ homeostasis: Na^+^ strongly inhibits K^+^ transport proteins such as AKT1 [7] and HAK5 [8] and therefore interferes with K^+^ uptake by root cells. Furthermore, the plasma membrane is depolarized by increased Na^+^ influx, which activates outwardly rectifying K^+^ channels such as GORK and enhances K^+^ efflux [9].

Because Na^+^ is very similar to K^+^ in terms of physiochemical properties such as ionic radius and ion hydration energy, its accumulation can inhibit many K^+^-requiring enzymes in the cytoplasm, which are involved in catalyzing various metabolic processes such as photosynthesis, starch formation and protein synthesis [3]. Such inhibition results from the competition between Na^+^ and K^+^ for the major binding sites in the enzymes. Therefore, maintaining K^+^/Na^+^ homeostasis in the cytosol is critical for enzyme activities and serves as an important factor in determining plant salt tolerance [10].

Extensive biochemical and genetic studies with the salt overly sensitive (SOS) *Arabidopsis* mutants have identified a molecular mechanism by which the model plant senses and responds to high salinity in soils [11]. CBL4 protein detects the cytosolic calcium signals elicited by the salt stress and subsequently activates the enzymatic activity of CIPK24 via direct interaction, which are respectively encoded by the genomic loci *SOS3* and *SOS2* [12,13,14]. The activated CIPK24 then phosphorylates the plasma membrane-localized SOS1 protein to promote the Na^+^/H^+^ antiporter activity, enhancing Na^+^ efflux from the root cells [15,16]. In addition, recent findings demonstrated that CIPK24 can associate with various other proteins to modulate salt tolerance [17,18,19]. Therefore, it is obvious that CIPK24 can serve as a key regulatory component of the salt stress response in *Arabidopsis*. As a matter of fact, this regulatory role of CIPK24 appeared to be conserved in other plants as well, because transgenic plants that overexpress tomato *CIPK24* (SlCIP24) showed enhanced tolerance to salt stress [20,21].

As a member of the SnRK3/CIPK family, CIPK24 is constituted of a serine/threonine protein kinase domain in the N-terminal end and an auto-inhibitory domain in the C-terminal end [22]. Because the C-terminal regulatory region blocks the active site in the kinase domain via intramolecular interaction, CIPK24 is not constitutively active in phosphorylating substrate protein [23]. This auto-inhibitory regulation is abolished by Ca^2+^-bound CBL4 that physically associates with the NAF/FISL motif in the C-terminal region of CIPK24, allowing the kinase domain to be free and gain the substrate phosphorylation activity [12]. Interestingly, CIPK24 could be activated by another mechanism involving phosphorylation of the threonine (Thr) residue at the 168th position from the N-terminus, which is located within the activation loop of the kinase domain [24].

Based on these regulatory mechanisms, two constitutively active CIPK24 mutant forms that are independent of CBL4 were generated in *Arabidopsis* by either deleting the auto-inhibitory C-terminal region harboring the CBL4-binding motif or substituting the 168th Thr to aspartate (Asp) to mimic phosphorylation in the activation loop. Moreover, the simultaneous introduction of the two mutations resulted in a synergistic effect (also known as a superactive form), further activating the kinase activity of CIPK24 [15,24]. In this study, we have identified and cloned two tomato genes, designated SlCBL4 and SlCIPK24, which are similar to *Arabidopsis* CBL4 and CIPK24 genes, respectively. Biochemical and cellular analyses have demonstrated that these two tomato genes retain the same biological function in salt stress response, as in the case of the *Arabidopsis* genes.

## 2. Results

### 2.1. Isolation and Sequence Analysis of SlCBL4 and SlCIPK24 cDNA

**Figure 1:**
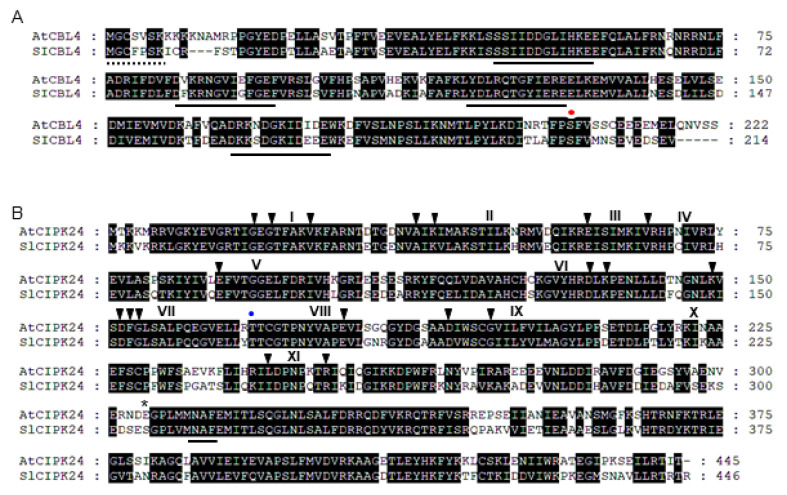
Sequence analysis of SlCBL4 and SlCIPK24.

We have performed BLAST searches on the tomato (*Solanum lycopersicum*) genome (Sol Genomics Network) using *AtCBL4* and *AtCIPK24* cDNA as query sequences and identified tomato homologs designated as *SlCBL4* (AB675686) and *SlCIPK24* (AJ717348), respectively. Based on the nucleotide sequences, we designed gene-specific primer sets and isolated via RT-PCR the full-length cDNA of the *SlCBL4* and *SlCIPK24* genes from tomato mRNAs. Sequence analysis indicated that the *SlCBL4* cDNA clone encodes a polypeptide of 214 amino acid residues sharing 73% identity with AtCBL4 (Figure 1A). Like AtCBL4, SlCBL4 possesses four EF-hand Ca^2+^-binding motifs [25], the N-myristoylation motif (MGXXXSK) mediating membrane association [26], and the conserved serine residue in the C-terminus [27]. As shown in Figure 1B, SlCIPK24 consists of 446 amino acid residues displaying 72% identity with AtCIPK24. The NAF motif [28], which is a common feature of all CIPK family members and mediates interaction with CBLs, is also found in the C-terminal region of SlCIPK24.

Alignments were performed with the Lasergene MegAlign Program (DNASTAR) and modified with the GeneDoc software. Identical amino acids are shaded black. Dashes represent gaps to maximize the alignment. (A) Amino acid sequence alignment of AtCBL4 (AT5G24270) and SlCBL4 (AB675686). The consensus N-myristoylation and EF-hand motifs were underlined with dashed and solid lines, respectively. The red dot indicates the conserved serine phosphorylation site. (B) Amino acid sequence alignment of AtCIPK24 (AT5G35410) and SlCIPK24 (AJ717348). Arrowheads and Roman numerals above the sequences indicate the conserved amino acids and subdomains of serine-threonine protein kinases, respectively. The blue dot and asterisk indicate 168th and 304th amino acid residues, respectively. The NAF motif was underlined.

### 2.2. SlCBL4 Interacts with SlCIPK24 in the Yeast Two-Hybrid System

Sequence analysis above showed that SlCBL4 and SlCIPK24 are very similar to *Arabidopsis* counterparts. Therefore, we first investigated whether SlCBL4 interacts with SlCIPK24 in the yeast two-hybrid system. To this end, the complete coding regions of SlCBL4 and SlCIPK24 were respectively cloned in the DNA-binding domain (pGBT⋅BS; BD) and GAL4 activation domain (pGAD⋅GH; AD) vectors, producing BD⋅SlCBL4 and AD⋅SlCIPK24. These BD and AD constructs were introduced into the yeast strain Y190 cells, and interaction between the two proteins was determined by analyzing expression of the reporter genes, imidazole glycerol-phosphate dehydratase (*HIS3*) and β-galactosidase (*LacZ*).

**Figure 2:**
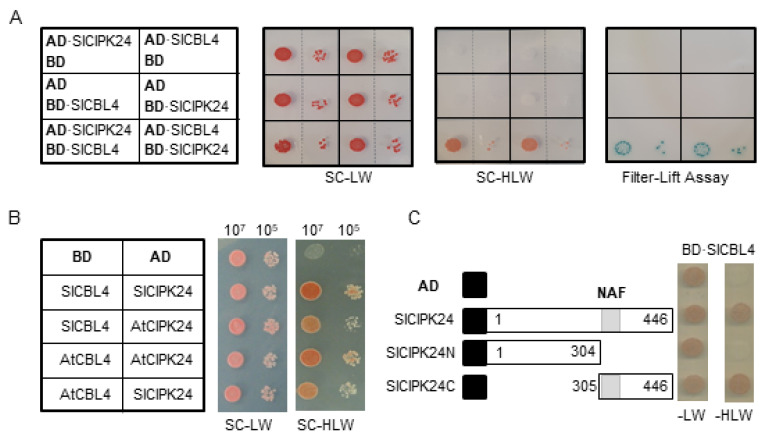
Yeast two-hybrid assays. (**A**) Vector swapping assay. The first panel at left shows the arrangement of the Y190 yeast cells harboring the indicated BD and AD plasmids. The second and third panels display yeast growth on synthetic complete (SC) medium lacking Leu and Trp (SC-LW) and SC medium lacking His, Leu and Trp (SC-HLW), respectively. The last panel shows β-galactosidase activity. (**B**) Comparative interaction analysis. The first panel at left represents the arrangement of the Y190 yeast cells harboring the indicated BD and AD plasmids. Co-transformed yeast cells were cultured, serially diluted, and spotted onto the indicated media. (**C**) Deletion analysis. SlCIPK24 deletion mutants were created in the AD vector and co-transformed into the yeast cells with BD·SlCBL4. Black boxes indicate the activation domain of GAL4 transcription factor. Numbers in the white boxes represent the beginning and the ending positions of each protein fragment. The gray box designates the NAF motif spanning from 307 to 329 amino acids.

As shown in Figure 2A (left column), the yeast cells carrying both BD⋅SlCBL4 and AD⋅SlCIPK24 grew well on the selection medium (SC-HLW) and developed a blue color in the filter-lift assay, indicating expression of the *HIS3* and *LacZ* reporter genes. In contrast, the control cells possessing either AD⋅SlCIPK1/BD or AD/BD⋅SlCBL4 plasmid combinations did not express the reporter genes. These results clearly suggest that SlCBL4 can interact with SlCIPK24 as in the case of *Arabidopsis* CBL4 and CIPK24. Furthermore, vector-swapping analysis using AD⋅SlCBL4 and BD⋅SlCIPK24 constructs demonstrated that the SlCBL4-SlCIPK24 interaction is independent of the cloning vectors (Figure 2A, right column). We also tested whether SlCBL4 and SlCIPK24 can interact with their *Arabidopsis* counterpart. As shown in Figure 2B, both SlCBL4 and SlCIPK24 exhibited interaction affinity toward AtCIPK24 and AtCBL4, respectively, in the yeast two-hybrid system. However, it should be noted that these heterologous interactions occurred at a slightly lower strength compared to the SlCBL4-SlCIPK24 interaction.

Since it is well known that the NAF motif is required and sufficient for the *Arabidopsis* CBL-CIPK interaction [24,28,29], we examined its importance in the interaction between SlCBL4 and SlCIPK24. For this purpose, we made two deletion constructs of SlCIPK24, AD⋅SlCIPK24N and AD⋅SlCIPK24C (Figure 2C). As expected, yeast two-hybrid assays showed that the SlCIPK24N mutant lacking the NAF motif failed to interact with SlCBL4, while SlCIPK24C retained the interaction. Taken together, SlCBL4 and SlCIPK24 appear to interact with each other using a structural platform very similar to *Arabidopsis* CBL4 and CIPK24.

### 2.3. SlCBL4 Physically Associates with SlCIPK24 in a Ca^2+^-Dependent Manner

**Figure 3:**
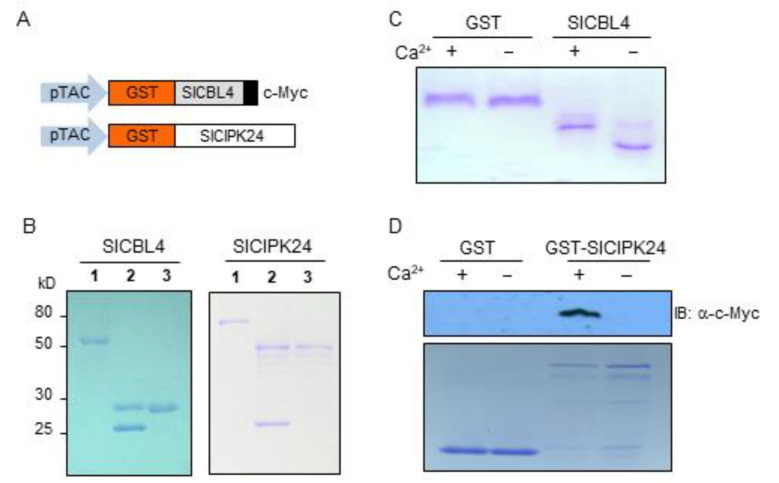
Recombinant SlCBL4 protein possesses Ca^2+^-binding activity and interact with SlCIPK24 in vitro. (**A**) Schematic diagrams of pGEX-SlCBL4:c-Myc and pGEX-SlCIPK24 constructs. (**B**) Expression and purification of the recombinant SlCBL4 and SlCIPK24 proteins. Lanes 1 to 3 contain the GST-fused forms, the thrombin-digested forms, and purified forms, respectively. The proteins were analyzed by SDS-PAGE and stained with Coomassie Brilliant Blue. The molecular masses of the proteins are indicated at left in kilodaltons (kD). (**C**) Gel mobility shift assay showing Ca^2+^-binding activity of the SlCBL4:c-Myc protein. The recombinant SlCBL4:c-Myc protein was incubated in EGTA-(-) or calcium-containing buffer (+) before being analyzed by 12% native PAGE. GST protein was used as a negative control. (**D**) Pull-down assay demonstrating a Ca^2+^-dependent SlCBL4-SlCIPK24 association. The GST-SlCIPK24 fusion protein was used as a bait to retrieve the prey SlCBL4:c-Myc in the presence (+) or absence (-) of calcium. For a negative control, the GST was used as bait. Top panel is an immunoblot probed with mouse anti-c-Myc antibody. Bottom panel is a Coomassie Blue-stained SDS-PAGE gel showing the amount of the bait proteins used in the assay.

Since SlCBL4 has the typical EF-hand Ca^2+^-binding motifs, we investigated whether the SlCBL4 protein can actually bind Ca^2+^ in vitro. To this end, the recombinant SlCBL4 protein with the c-Myc tag was constructed (Figure 3A) and purified from *E. coli* using the glutathione S-transferase (GST) gene fusion system. As shown in Figure 3B (left column), SlCBL4:c-Myc protein (~27 kD) was initially retrieved as a GST-fused form (~52 kD) and subsequently subjected to thrombin digestion to obtain the GST-free form. In the gel mobility shift assay [30], the SlCBL4 protein migrated at a slower rate in the lane with Ca^2+^ than in the lane without Ca^2+^ (Figure 3C). However, the GST control protein did not show such mobility shift. Therefore, we can conclude that SlCBL4 is a functional Ca^2+^-binding protein.

We also performed the pull-down assay as shown in Figure 3D to confirm the SlCBL4-SlCIPK24 interaction in the yeast two-hybrid system and to investigate whether Ca^2+^ has any effect on this interaction. The bait proteins GST and GST-SlCIPK24 were respectively mixed with the prey protein SlCBL4 tagged with c-Myc in the presence or absence of Ca^2+^ and determined whether GST beads can pull down the prey (see Methods). As shown in Figure 3D, the Ca^2+^-binding protein SlCBL4 was efficiently retrieved by the GST-SlCIPK24 bait protein only in the presence of Ca^2+^. In contrast, GST alone did not retrieve SlCBL4 regardless of Ca^2+^. Therefore, it is clear that SlCBL4 physically associates with SlCIPK24 in a Ca^2+^-dependent manner.

### 2.4. SlCBL4 and SlCIPK24 Interact at the Plasma Membrane of Plant Cells

**Figure 4:**
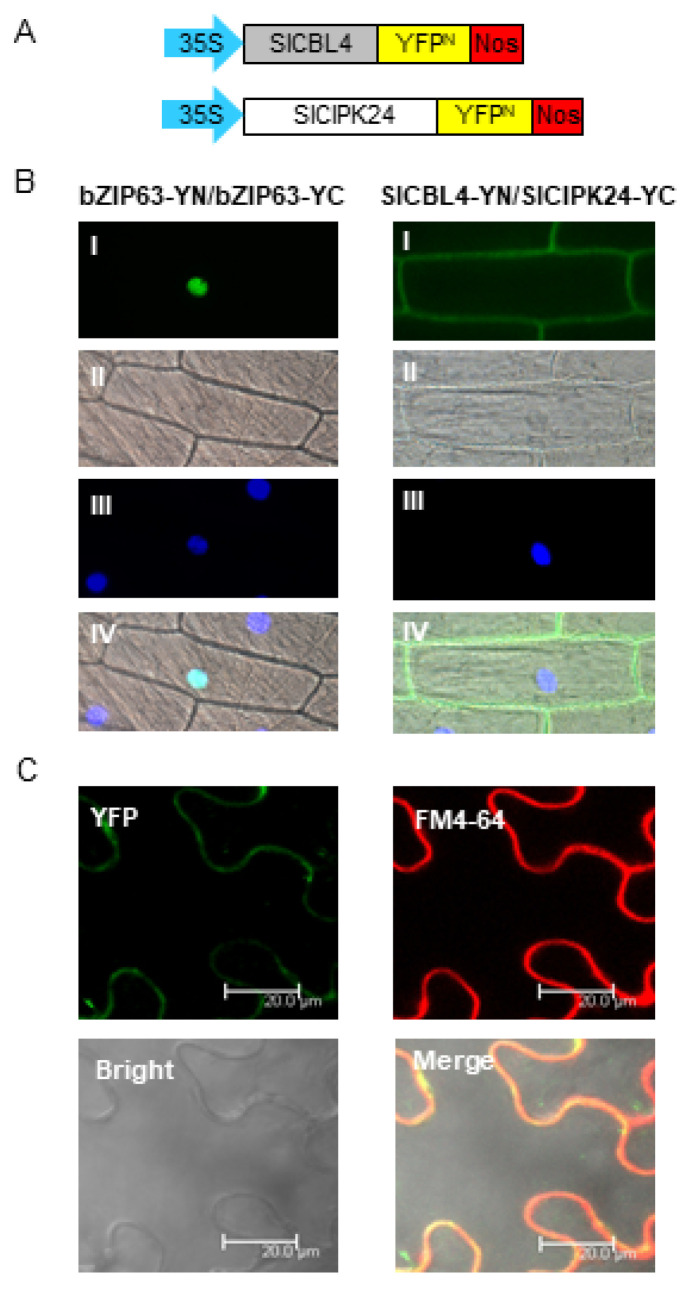
In vivo interaction between SlCBL4 and SlCIPK24. (**A**) Schematic diagrams of SlCBL4-YN (**top**) and SlCIPK24-YC (**bottom**) constructs. (**B**) Bimolecular fluorescence complementation (BiFC) analysis in onion epidermal cells. The indicated plasmids were introduced into the plant cells via the biolistic particle-delivery system. The bZIP63-YFP^N^ and bZIP63-YFP^C^ plasmids were used as a positive control. Following 18-h incubation at 23 °C, the onion cells were analyzed with a fluorescence microscope. I, II and III show images of yellow fluorescence, bright field, and nuclei visualized by 4,6-diamidino-2-phenylindole (DAPI) staining, respectively. IV shows the merged image. (**C**) Images of tobacco (*Nicotiana benthamiana*) leaf epidermal cells infiltrated with Agrobacterium (GV3101) carrying the SlCBL4-YN and SlCIPK24-YC constructs. YFP, FM4-64 and Bright indicate images of YFP, FM4-64 and bright field, respectively. Merge show their merged image.

To further verify the physical interaction between SlCBL4 and SlCIPK24 in vivo, we performed the bimolecular fluorescence complementation (BiFC) assay [31]. We made SlCBL4-YN and SlCIPK24-YC chimeric constructs by fusing SlCBL4 and SlCIPK24 to the N-terminal YFP fragment (YN) and the C-terminal YFP fragment (YC) in the plant expression vectors, respectively (Figure 4A). These constructs were then transiently expressed in onion epidermal cells via particle bombardments. As shown in Figure 4B, YFP fluorescence was mostly detected at the plasma membrane of the onion cells expressing the two fusion proteins, while the plant cells expressing the control construct bZIP63-YN and bZIP63-YN displayed the signal only in the nucleus. The *Arabidopsis* basic leucine zipper (bZIP) transcription factor bZIP63 (At5g28770) is known to form a homodimer in the nucleus of plant cells [32]. We further examined the SlCBL4-SlCIPK24 interaction in tobacco leaf epidermal cells. The two chimeric constructs were introduced into the plant cells via the *Agrobacterium*-infiltration method. As shown in Figure 4C, the fluorescent signals from YFP and the amphiphilic styryl dye FM4-64 overlapped at the plasma membrane. These fluorescent images clearly demonstrated that SlCBL4 and SlCIPK24 can actually interact with each other in vivo and the physical interaction predominantly occurs at the plasma membrane.

### 2.5. Expression Patterns and Subcellular Localization of SlCBL4 and SlCIPK24

**Figure 5:**
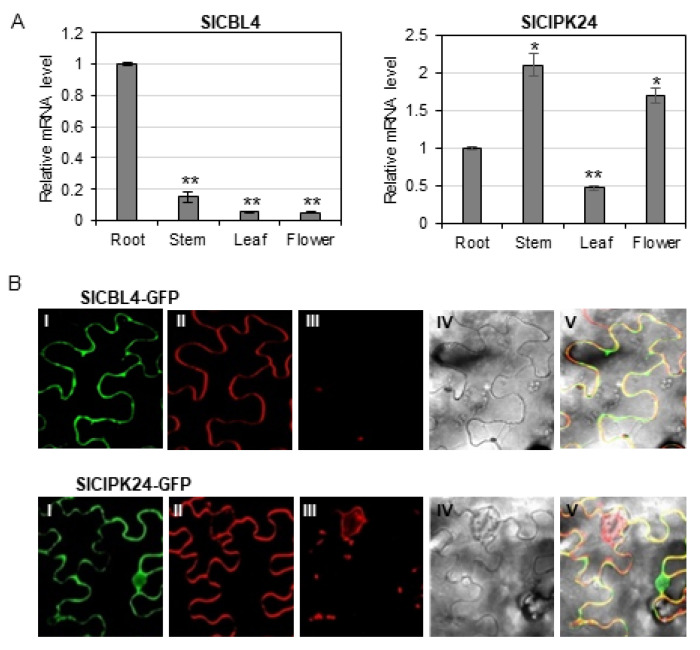
Expression patterns and subcellular localization of SlCBL4 and SlCIPK24. (**A**) Expression patterns of *SlCBL4* and *SlCIPK24* in different tomato organs using RT-qPCR analysis. The expression levels of *SlCBL4* and *SlCIPK24* in roots were set to 1.0 to show relative abundance differences. Tomato *actin7* (NM 001308447.1) and *β-tubulin* (Solyc04g081490.2.1) genes were employed as internal control. Error bars denote standard deviation (SD) of three biological replicates. Significance was calculated using the Student’s *t*-test: * *p* < 0.05, ** *p* < 0.01. (**B**) Tobacco (*Nicotiana benthamiana*) leaf epidermal cells infiltrated with Agrobacterium (GV3101) carrying the indicated plasmids. I, II, III and IV show images of GFP, FM4-64, auto-fluorescence and bright field, respectively. V displays the merged image.

Our analyses above clearly showed that SlCBL4 and SlCIPK24 can interact with each other when they coexist. Therefore, it is necessary to investigate gene expression patterns and subcellular localization of SlCBL4 and SlCIPK24 in order to determine whether the interaction between the two proteins can actually occur in vivo in tomato plants. To perform RT–qPCR analysis, we extracted total RNA from various parts of 7-week-old tomato plants (cv. Moneymaker), which include roots, stems, leaves, and flowers. As shown in Figure 5A, both SlCBL4 and SlCIPK24 genes were relatively well expressed in the roots, although their expression patterns and levels are generally different from each other: *SlCBL4* transcript levels were less expressed in other organs compared to its expression in roots, whereas *SlCIPK24* showed higher expression in stems and flowers than in roots. It should be noted that *SlCIPK24* was expressed more strongly in all organs tested than *SlCBL4*. In any case, it is clear that SlCBL4 and SlCIPK24 are expressed in the same organs, at least in tomato roots.

Although our BiFC assay tends to show that SlCBL4 and SlCIPK24 can form a complex at the plasma membrane of onion epidermal cells when they were co-expressed, we further investigated their subcellular localization in tobacco leaf cells. To perform this experiment, we first constructed SlCBL4-GFP and SlCIPK24-GFP fusion genes, which were driven by the cauliflower mosaic virus *35S* promoter. These constructs were then separately and transiently expressed in tobacco *(Nicotiana benthamina*) leaves via *Agrobacterium* (GV3101) infiltration. According to the confocal fluorescence images in Figure 5B, SlCBL4 that harbors the N-myristoylation motif was almost exclusively localized to the plasma membrane, such as *Arabidopsis* CBL4 [33]. However, SlCIPK24 appeared to be localized throughout the plant cell, including the plasma membrane and nuclear compartments. Overall, these results clearly indicate that SlCBL4 and SlCIPK24 have some overlapping spatial expression patterns, and therefore it is likely that they can form a complex at the plasma membrane of tomato root cells.

### 2.6. Overexpression of SlCBL4 or Superactive SlCIPK24 Mutant Confers Salt Tolerance to Transgenic Tomato Plants

To determine whether SlCBL4 and SlCIPK24 play a role in salt stress response, we generated transgenic tomato (cv. Moneymaker) plants overexpressing SlCBL4 or SlCIPK24M (a constitutively superactive SlCIPK24 mutant form). As mentioned earlier, SlCIPK24M was created by changing the 168th amino acid Thr to Asp in the C-terminal deleted SICIPK24 mutant lacking the self-inhibitory NAF/FISL motif [34]. Three independent T0 transformants were selected and self-pollinated for two generations to obtain homozygous transgenic lines. We determined the mRNA levels of the introduced genes by real-time RT-qPCR analysis and found a couple of transgenic lines that show significantly increased transcript levels compared with the wild-type (WT) tomato plants (Supplemental Figure S1). Among the transgenic plants, the *SlCBL4-OX2* and *SlCIPK24M-OX1* lines were selected for further analysis, because they displayed higher expression levels than others.

**Figure 6:**
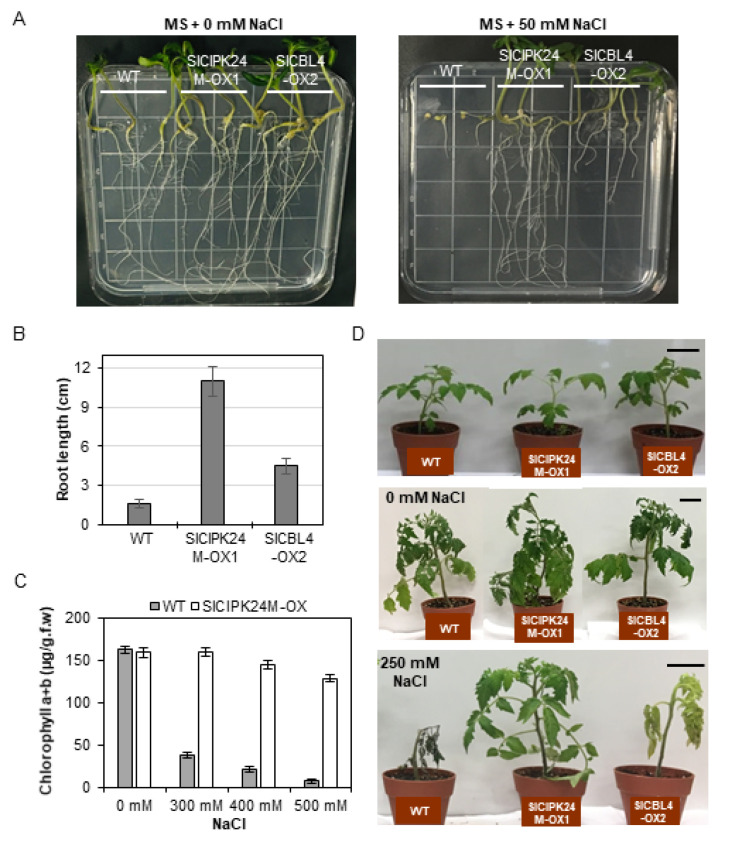
Phenotype analysis of transgenic tomato plants under salt stress. (**A**) Vertical growth assay. The indicated seeds were plated on MS agar media supplemented with 0 mM (**left** panel) or 50 mM NaCl (**right** panel), which were then placed vertically in the growth chamber. The photos were taken on the 13th day after transfer. (**B**) Root length of the seedlings from the experiment described in (**A**). The seedlings on 50 mM NaCl plates were measured. (**C**) Leaf chlorophyll contents. Ten-day-old soil-grown seedlings were treated with the different concentrations of NaCl solution for two days. For each condition, four plants were analyzed. Error bars denote standard deviation (SD) of three biological replicates. Values are significantly different (the Student’s *t*-test, *p* ≤ 0.05). (**D**) Salt stress tolerance of the mature transgenic plants. Three-week-old plants (**top**) were treated with 0 mM (**middle**) or 250 mM NaCl solution (**bottom**). The photos were taken on the 21st day after treatment. *Scale bars,* 5 cm. All the experiments were performed in three biological replicates.

We first performed the vertical growth assay to analyze the plant response to salt stress at seedling stages by sowing the seeds of WT, *SlCBL4-OX2*, and *SlCIPK24M-OX1* on MS agar plates supplemented with 0 or 25 mM NaCl (Figure 6A). On the MS medium without salt, the two transgenic plants displayed no significant differences in growth rate compared with WT (Figure 6A, left panel). On the salt-containing MS medium, however, both transgenic lines showed much better growth rate than WT (Figure 6A, right panel). In particular, SlCIPK24-OX1 exhibited dramatically enhanced tolerance to the salt stress. The root lengths of the SlCIPK24M-OX1 and SlCBL4-OX2 transgenic plants were approximately 7.3 times and 2.4 times longer than that of the WT plants, respectively (Figure 6B).

As summarized in Figure 6C, WT seedlings rapidly and drastically lost chlorophylls when exposed to high concentrations of NaCl from 300 mM to 500 mM. However, such a decrease in the chlorophyll content was not detected in SlCIPK24-OX2 seedlings. We further expanded the salt tolerance assay to the adult stages by supplying a 250 mM NaCl solution to the 3-week-old plants every three days. Following 21 days of the treatment, WT plants died after severe growth arrest, whereas SlCIPK24M-OX2 plants continued to grow well without any significant symptoms (Figure 6D). The salt tolerance levels of SlCBL4-OX1 plants are somewhat in between those of WT and SlCIPK24M-OX2 plants: The SlCBL4-OX1 plants grew much better than WT plants under the salt stress condition, but the leaves turned yellow over time and eventually fell off. Without the salt stress, however, there were no discernable phenotypic differences among all three plant lines. Similar results were also obtained with SlCBL4-OX3 and SlCIPK24M-OX2 (Supplemental Figure S2). Overall, these results indicate that overexpression of SlCBL4 or SlCIPK24M can confer enhanced salt tolerance in tomato plants. It also strongly suggests that the SlCBL4 and SlCIPK24 complex may play an important role in the salt stress response of tomato plants.

### 2.7. SlCIPK24M-Overexpressing Plants Display Higher Expression Levels of Stress Genes

Since alterations in the stress tolerance of plants are often accompanied by alterations in expression of the stress genes [34,35], we investigated whether such changes actually occur in the SlCIPK24M-overexpressing plants. To this end, we selected three stress marker genes (SlRD22, *SlRD29B and TAS14*) in tomato and monitored their expression patterns. In *Arabidopsis*, expression of the *RD22* and *RD29B* genes was induced by high salt and drought, and their mRNA levels were known to correlate with the plant stress tolerance [36,37]. *TAS14* is a tomato dehydrin gene that can serve as a salt stress-inducible marker [38,39].

**Figure 7:**
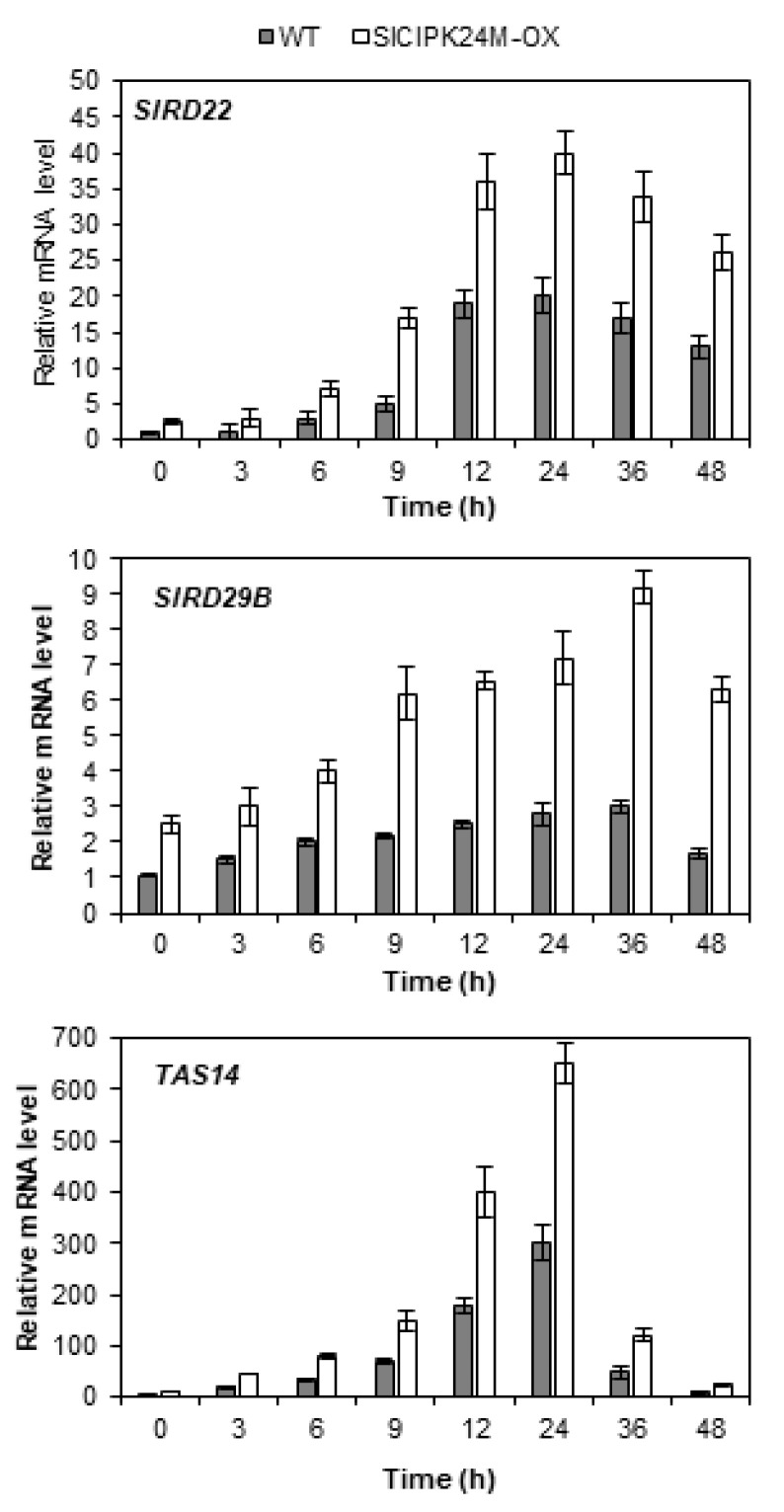
Expression levels of Stress Marker Genes in WT and SlCIPK24M-overexpressing tomato plants under 200 mM NaCl stress at different times. Eleven-day-old plants were treated with 200 mM NaCl for the indicated time period. The expression levels of *SlRD22* (Solyc08g068150.4.1), *SlRD29B* (Solyc03g025810.4.1) *and TAS14* (X51904.1) in WT at 0 h were set to 1.0 to show relative abundance differences. The tomato *actin7* and *β-tubulin* genes were employed as internal control. Error bars denote standard deviation (SD) of the means produced from three independent experiments. Values are significantly different (the Student’s *t*-test, *p* ≤ 0.05).

As shown in Figure 7, RT-qPCR analysis demonstrated that the transcript levels of *SlRD22* and *SlRD29B* were significantly induced by the high salt treatment in the WT tomato plants, indicating that these genes can be used as salt stress markers as in *Arabidopsis*. As anticipated, the *TAS14* gene, already known as a salt-inducible marker in tomato plants, was also highly induced, confirming the previous reports [38,39]. Interestingly, compared to the WT plants, the SlCIPK24M-overexpressing plants already accumulated substantial levels of the transcripts prior to exposure to the salt stress. On top of this high background level, the stress treatment further increased the expression levels of all the three stress marker genes in the transgenic tomato plants. These results clearly suggest that the SlCIPK24M-overexpressing plants have altered signaling pathways not only under normal conditions, but also under salt stress conditions.

### 2.8. Tomato Plants Overexpressing SlCIPK24M Accumulate Higher Na^+^ and K^+^ Contents in the Roots Than WT Plants

**Figure 8:**
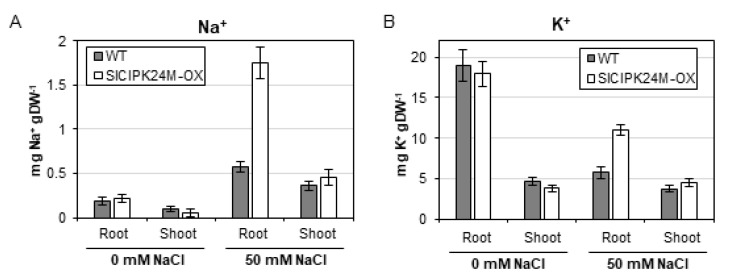
Na^+^ (**A**) and K^+^ (**B**) contents in roots and shoots of wild-type and SlCIPK24M-overexpressing tomato plants. Eleven-day-old seedlings were treated with 0 or 50 mM NaCl solution (1/6 MS) for five additional days. For each condition, three plants were harvested and pooled in roots and shoots for the measurement of Na^+^ and K^+^ contents. Error bars denote standard deviation (SD) of three biological replicates. Values are significantly different (the Student’s *t*-test, *p* ≤ 0.05).

To better understand how SlCIPK24M overexpression confers salt tolerance in the transgenic tomato plants, we measured Na^+^ and K^+^ contents in the root and shoot. As illustrated in Figure 8, under normal condition without NaCl supplementation, both plants displayed similar patterns of Na^+^ and K^+^ accumulation in the two plant organs. However, when treated with 50 mM NaCl solution, the SlCIPK24M-OX1 plants contained significantly higher levels of Na^+^ and K^+^ concentrations in the root than WT plants. Interestingly, no such difference was found in the shoot in that Na^+^ and K^+^ ions were detected at similar levels in both plants. Taken together, our data suggest that the enhanced salt tolerance of the transgenic plants overexpressing the superactive SlCIPK24 (SlCIPK24M) is associated with the increased deposition of Na^+^ and K^+^ in the root under salt stress.

## 3. Discussion

Salt tolerance of plants is a multigenic trait and therefore it can be achieved by a variety of cellular responses, which include Na^+^ exclusion and vacuolar sequestration, K^+^/Na^+^ homeostasis, osmotic adjustment, regulation of membrane potential, and activation of antioxidant enzymes [4]. According to recent studies, mechanosensitive Ca^+^ channels and Ca^+^-dependent signaling pathways are involved in sensing and responding to salt stress in plants [40,41]: Soil salinity stimulates the Ca^+^ channels to generate distinct Ca^+^ signatures, which are subsequently perceived and transduced by calcium sensors such as calmodulins, calcium-dependent protein kinases (CDPKs) and CBL proteins to regulate various targets such as enzymes, channels and transcription factors [42,43]. Among the calcium sensors, CBL4 (also known as SOS3) is the most extensively investigated member of the *Arabidopsis* CBL family so far and provides an exemplary molecular mechanism how Ca^+^-binding proteins can mediate the salt stress response in plants [11]. As a sensor relay protein, CBL4 undergoes a conformational change upon Ca^+^ binding and stimulates the interaction partner CIPK24 (aka SOS2) to phosphorylate the SOS1 Na^+^/H^+^ antiporter. The phosphorylated SOS1 protein becomes active and remove excess Na^+^ from the cytosol to other compartments [15,16]. Exploiting the regulatory mechanism in this SOS signaling pathway, we attempted in this study to produce tomato plants with enhanced salt tolerance. To this end, two tomato genes showing sequence homology to *Arabidopsis* CBL4 and CIPK24 were isolated and named SlCBL4 and SlCIPK24, respectively. Then, these homologous genes were subjected to a series of analyses to determine whether they are really involved in mediating salt tolerance in tomato plants.

First of all, our yeast two-hybrid assays demonstrated that SlCBL4 and SlCIPK24 interact with each other just like the *Arabidopsis* CBL4 and CIPK24 proteins. We also found that SlCBL4 and SlCIPK24 can associate respectively with *Arabidopsis* CIPK24 and CBL4, revealing structural similarities between them. Next, we showed that SlCBL4 is actually a Ca^2+^-binding protein and is able to make a complex with SlCIPK24 in a Ca^2+^-dependent manner via the in vitro assays. Interestingly, this Ca^2+^-dependent interaction was not observed in the *Arabidopsis* counterparts: CBL4 and CIPK24 physically interacted with each other in the absence of Ca^2+^, even though Ca^2+^ was still required for the *Arabidopsis* complex to become active and phosphorylate substrate proteins [12]. In this respect, it is noteworthy that another member of the *Arabidopsis* CBL family, CBL1, forms a complex with CIPK1 only in the presence of Ca^2+^ [44]. Judging from these results, the Ca^2+^ requirements for the CBL-CIPK complex formation itself can vary depending on the participating members and does not appear to be prerequisite for transmitting Ca^2+^ signals.

We also performed the BiFC assay on onion epidermal cells and confirmed that when SlCBL4 and SlCIPK24 are co-expressed, they can physically interact in the plants cells, primarily at the plasma membrane. Furthermore, their gene expression and subcellular localization patterns revealed that SlCBL4 and SlCIPK24 can be present simultaneously at the plasma membrane, notably of tomato root cells. These results together clearly indicated that the SlCBL4-SlCIPK24 complex could also be formed in planta, which is in good agreement with the previous finding obtained with *Arabidopsis* CBL4 and CIPK24 proteins [45].

Finally, our phenotypic analyses showed that the transgenic tomato lines that overexpress SlCBL4 or superactive form of SlCIPK24 mutant (SlCIPK24M) are more tolerant to salt stress regardless of their development stages (both seedlings and mature plants) than wild-type plants. Overall, our findings in this present work demonstrate that, like the *Arabidopsis* CBL4 and CIPK24 proteins [46,47], SlCBL4 and SlCIPK24 are also involved in mediating salt tolerance of tomato plants by sensing and transducing Ca^+^ signals elicited by high salinity stress. In this respect, the CBL-CIPK Ca^+^-signaling networks, initially identified in the model plant *Arabidopsis* [43], appear to be present in tomato plants as well and probably participate in mediating a variety of environmental stresses.

Somehow SlCIPK24M-overexpressing tomato plants displayed particularly strong salt tolerance, which was accompanied by higher expression levels of the stress genes such as *SlRD22*, *SlRD29B* and *TAS14*. Although we do not understand how these two events are related, it is clear that the superactive SlCIPK24M overexpression increased the expression of the stress genes in transgenic plants. It is actually consistent with the *Arabidopsis* studies, which demonstrated that *RD22* and *RD29B* mRNA levels were closely associated with stress tolerance [36,37]. However, it should be noted that the enhanced salt tolerance of SlCIPK24M-overexpressing plants is not necessarily the result of increased expression of the stress genes, although the phenotype is closely associated with the levels of their mRNA accumulation in the plants. Therefore, future scientific research is needed to find out the molecular mechanisms underlying these associations in plant cells.

When exposed to high salt conditions, the SlCIPK24M plants deposited higher concentrations of Na^+^ and K^+^ in the roots than WT plants, but not in the shoots. Initially, this result was somewhat perplexing to us because CIPK24 was well known to promote Na^+^ extrusion from the *Arabidopsis* root cells by activating the Na^+^/H^+^ antiporter activity of SOS1 at the plasma membrane [15,16]. Judging from the fact that tomato also contains a SOS1-like gene (named SlSOS1, Solyc01g005020.3.1), it is likely that SlCIPK24 may phosphorylate SlSOS1 and convert it to its active form. However, we soon realized that such physiological changes in the transgenic plants could be explained by the fact that CIPK24 can also target the tonoplast Na^+^/H^+^ antiporter and increases Na^+^ accumulation in the large central vacuole [48]. Furthermore, CIPK24 can indirectly affect the Na^+^ storage as well by interacting with and activating the vacuolar H^+^-ATPase to increase H^+^ concentrations in the vacuole [49]. When the concentration of H^+^ increases in the subcellular organelle, the vacuolar membrane-localized Na^+^(K^+^)/H^+^ antiporters (NHX1-4) can absorb more Na^+^/K^+^ from the cytosol in exchange for the vacuolar H^+^ ions, reducing the deleterious effect of cytosolic Na^+^ [2,3]. These findings clearly showed that salt tolerance is closely related with the regulation of Na^+^ and K^+^ homeostasis in plants. It is also noteworthy that maintaining a low Na^+^/Ca^+^ ratio in the tomato shoot apex is critical for the tissues to grow under salt stress [50]. In this regard, it is understandable that SlCIPK24M overexpression appears to confer salt tolerance in the transgenic tomato plants by keeping the cytosolic Na^+^ concentration at low levels by sequestering excess Na^+^ in the vacuole.

## 4. Materials and Methods

### 4.1. Plant Materials and RNA Preparation

*Solanum lycopersicum* (cv. Moneymaker) plants were grown on MS agar medium at 25 °C in a growth chamber under long-day conditions (16-h-light/8-h-dark cycle). Total RNA was extracted from the tomato plant tissues using the TRIzol reagent (Invitrogen, Waltham, MA, USA) according to the manufacturer’s instruction. Quality and quantity of the RNAs were monitored by spectrophotometry (OD: 260/280) and by electrophoresis in a 1.2% agarose gel.

### 4.2. First-Strand cDNA Synthesis and Isolation of SlCBL4 and SlCIPK24 cDNA

Total RNA was isolated from tomato whole seedlings grown on MS agar medium for 2 weeks and treated with 100 mM NaCl for 24h. Using SuperScript III First-Strand Synthesis System (Thermo Fisher Scientific, Seoul, Korea), first-strand cDNA was reverse-transcribed from 3 µg of the total RNA according to the manufacturer’s instructions. For isolation of SlCBL4 and SlCIPK24 cDNA, 2 µL of cDNA synthesized above was used as template for a 50-µL PCR reaction with an appropriate primer set (SlCBL4-1/SlCBL4-2, and SlCIPK24-1/SlCIPK24-2, respectively).

### 4.3. Yeast Two-Hybrid Assays

In this study, a Gal4p-based yeast two-hybrid assay system [51] was used. Genes were first cloned into either the DNA- binding domain (pGBT9.BS, BD) or the activation domain (pGAD.GH, AD) vectors, and then they were transformed into yeast Y190 strain by the lithium acetate method [52]. Yeast transformants carrying both BD and AD plasmids were selected on synthetic medium lacking Leu and Trp (SC-LW) and were subsequently plated on synthetic medium lacking His, Leu and Trp (SC-HLW) to determine expression of the *HIS3* nutritional reporter gene. The His^+^ yeast cells were further tested for the expression of the other reporter gene β-galactosidase by the filter-lift assay as described previously [53] to verify interaction.

### 4.4. Expression and Purification of GST Fusion Proteins

Recombinant GST-fused proteins were expressed and purified basically according to the protocols described in the GST gene fusion system (GE Healthcare, Chicago, IL, USA). Briefly, *Escherichia coli (E. coli)* BL21 cells carrying a GST fusion plasmid were cultured overnight at 37 °C and were subcultured until the OD_600_ reached approximately 0.55. To induce expression of the GST fusion protein, Isopropyl-β-D-thiogalactopyranoside (IPTG) was added to a final concentration of 50 μM. Following 3 h induction at 20 °C, the bacterial cells were collected by centrifugation, resuspended in ice-cold lysis buffer (50 mM Tris-HCl, pH 7.4, 100 mM NaCl, 1 mM PMSF, 5 mM DTT, 5 mM EDTA, and 1 mM EGTA), and ruptured by sonication. The cell lysate supplemented with 1% Triton X-100 was incubated for 1 h incubation on ice and was centrifuged at 10,000× *g* for 10 min. Glutathione-Sepharose 4B beads were used to retrieve the GST fusion proteins from the supernatant. The beads were washed five times with ice-cold washing buffer (50 mM Tris-HCl, pH 7.4, 100 mM NaCl). Thrombin protease (GE Healthcare) was used to remove GST from the fusion protein.

### 4.5. Gel-Shift Assays

SlCBL4 tagged with c-Myc (SlCBL4:c-Myc) was prepared from the recombinant GST-SlCBL4:c-Myc protein by removing GST through thrombin digestion. The purified SlCBL4:c-Myc protein was incubated on ice for 30 min in the washing buffer containing either 1 mM EGTA or 2 mM CaCl_2_ and was resolved by native polyacrylamide gel electrophoresis. GST protein alone was used as control. Protein bands were stained with Coomassie Brilliant Blue R-250 solution to monitor mobility patterns.

### 4.6. Pull-Down Assay and Immunoblot Analysis

Pull-down assay and immunoblot analysis were performed as described previously [54] with minor modification. Briefly, GST and GST-SlCIPK24 bait proteins attached to the glutathione-Sepharose 4B beads were incubated with SlCBL4:c-Myc prey protein at 4 °C for 1 h in the binding buffer (50 mM Tris-HCl, pH 7.4, 100 mM NaCl, 0.05% Tween 20, and 1 mM PMSF) containing either 1 mM CaCl_2_ or 2 mM EGTA. The beads were washed six times with 500 µL of the corresponding binding buffer. Pull-down samples were separated by SDS-PAGE, transferred onto polyvinylidene fluoride (PVDF) membranes (Millipore), and subjected to immunoblot analysis. The mouse anti-c-Myc (9E10) (Santa Cruz Biotechnology, Dallas, TX, USA) and goat anti-mouse IgG (H + L) conjugated with horseradish peroxidase (Invitrogen) were used as the primary and secondary antibodies, respectively.

### 4.7. Bimolecular Fluorescence Complementation (BiFC) Assays and Subcellular Localization of GFP Fusion Proteins

BiFC constructs were introduced into the onion (*Allium cepa*) epidermal cells via particle bombardment [55] and GFP fusion constructs were infiltrated into tobacco (*Nicotiana benthamiana*) leaves using *Agrobacterium tumefaciens* strain GV3101 as described [56]. The plant cells transiently expressing the fluorescence proteins were analyzed with a confocal laser scanning microscope (LSM 510 META; Carl Zeiss, Overkochen, Germany) and Zeiss LSM 510 software (Zeiss LSM Image Examiner, Overkochen, Germany). Nuclei were stained for 5 min at room temperature with 1 µg mL^−1^ 4′, 6-diamidino-2-phenylindole (DAPI; Sigma-Aldrich, St. Louis, MO, USA) in phosphate-buffered saline solution and examined with a 417- to 477-nm band-pass filter. YFP images were captured with a 530- to 600-nm band-pass filter following excitation with an argon laser at 514 nm. GFP excitation was carried out with an argon laser at 488 nm, and emission was detected with a 515- to 565-nm band-pass filter.

### 4.8. Real-Time RT-qPCR Analysis

Real-time quantitative RT-PCR (RT-qPCR) was performed on the Rotor-Gene Q (Qiagen, Hilden, Germany) using the QuantiTect SYBR Green RT-PCR kit (Qiagen, Hilden, Germany) as described previously [53]. Briefly, 1 µg of total RNA extracted from plant tissues with the TRIzol reagent (Invitrogen) was used as template in a 10-µL reaction sample containing 0.25 µM each primer and 2x QuantiTect SYBR Green RT-PCR Master Mix and QuantiTect RT Mix. Following reverse transcription at 50 °C for 20 min, samples were denatured at 95 °C for 15 min and then subject to 35 PCR cycles consisting of 95 °C denaturation for 15 s, 55 °C annealing for 20 s, and 72 °C extension for 20 s. The tomato housekeeping genes *SlActin7* and β-tubulin (Solyc04g081490.2.1) were always co-amplified by a pair of forward and reverse primers and used as internal normalization controls. Primers for β-tubulin gene were designed according to Fernandez et al. [57].

### 4.9. Generation of Transgenic Tomato Plants

To make transgenic tomato plants that overexpress SlCBL4 or SlCIPK24M, the pATC-SlCBL4 and pATC-SlCIPK24M constructs (see below) were respectively introduced into *Agrobacterium tumefaciens* strain LBA4404 by the freeze-thaw method and used for transforming tomato (*Solanum lycopersicum*, cv. Moneymaker) plants. The transformation procedure was carried out basically as described by Park et al. [58].

Briefly, the tomato seeds were sterilized and germinated in a growth chamber at 25 °C under a 16 h/8 h light/dark cycle on MS medium [59], pH 5.8, supplemented with 3% sucrose and 0.8% phyto agar. Cotyledon explants of 12~13 day-old seedlings were pre-cultured for one day and then subjected to co-cultivation with an *Agrobacterium* culture for 3 days. Following co-cultivation, the explants were washed and placed upside down on selection medium (pH 5.8) containing 4.3 g L^−1^ MS salts, 3% sucrose, 1 mg L^−1^ zeatin, 0.1 mg L^−1^ IAA, 250 mg L^−1^ cefotaxime, 50 mg L^−1^ kanamycin, and 0.58% phyto agar. Shoots emerged from the explants were excised and transferred to rooting medium (pH 5.8) consisting of 2.15 g L^−1^ MS salts, 3% sucrose, 1 mg L^−1^ of IAA, 25 mg L^−1^ kanamycin, and 0.58% phyto agar. Tomato plantlets with roots were transplanted to soil and grown in a greenhouse to produce seeds. Seeds were harvested from these plants and screened on 1/2 MS medium containing 0.8% (*w*/*v*) phyto agar, 112 mg L^−1^ Gamborg’s B5 vitamin mixture, and 50 μg L^−1^ kanamycin. Homozygous transgenic lines were isolated and used for further analysis.

### 4.10. Salt Tolerance Analysis

Vertical root growth assay was performed as follows: Four seeds each of the wild-type (Moneymaker) and overexpression transgenic tomato lines (SlCBL4 and SlCIPK24M) were planted on MS agar plates supplemented with 0 mM or 50 mM NaCl. These plates were prepared in three biological replicates and incubated at 4 °C for 3 days before being placed vertically at 25 °C under long-day conditions (16-h-light/8-h-dark cycle). After 13 days of incubation, the root length of the seedlings was measured. Salt tolerance of the mature tomato plants were evaluated by treating 3-week-old soil-grown plants with 0 mM or 250 mM NaCl solution for additional 21 days.

### 4.11. Chlorophyll Measurement

The chlorophyll content in leaves was determined as described by Strain et al. [60]. Briefly, two leaves per tomato plant were collected and ground in liquid nitrogen. To extract chlorophyll, 3 volumes of 80% (*v*/*v*) acetone containing 1 μM KOH was added to the ground tissue and centrifuged at 16,000× *g* for 2 min. The supernatants were analyzed with the spectrophotometric method.

### 4.12. Measurement of Na^+^ and K^+^ Ions

Eleven-day-old tomato seedlings grown on 45° slanted MS agar medium with 3% sucrose and 56 mg L^−1^ Gamborg B5 vitamins in Magenta box were exposed to 70 mL 1/6 MS solution containing either 0 mM or 50 mM NaCl for 5 days. The seedlings were harvested, washed three times with deionized water, and separated in shoots and roots. Following 48 h drying at 80 °C, the samples were milled to powder and digested in a concentrated HNO_3_:HClO_4_ (2:1, *v*/*v*) solution. Na^+^ and K^+^ concentrations were determined by inductively coupled plasma atomic emission spectrophotometer (ICP-AES; Agilent Technologies, Santa Clara, CA, USA) in Korea Basic Science Institute.

### 4.13. Site-Directed Mutagenesis

The 168th amino acid threonine (T) in SlCIPK24 was mutated to aspartate (D) by changing the codon from ACC to GAC. The mutation was carried out with pGAD⋅CIPK24 plasmid template and a pair of primers (SlCIPK24-TD1 and SlCIPK24-TD2) using the QuikChange II Site-Directed Mutagenesis Kit (Agilent Technologies, Santa Clara, CA, USA) according to the manufacturer’s instructions. The resulting mutated plasmid was confirmed with DNA sequencing and designated as pGAD⋅CIPK24T168D.

### 4.14. Construction of Plasmids

The following plasmids were produced as described previously; pGBT⋅AtCBL4 [46], pGAD⋅AtCIPK24 [22], bZIP63-YFP^N^ and bZIP63-YFP^C^ [31]. The pGBT⋅SlCBL4 and pGAD⋅SlCBL4 plasmids were created by cloning the coding region of *SlCBL4* cDNA, amplified from the first-strand cDNAs with SlCBL4-1 and SlCBL4-2 primers, into the into *EcoR*I/*Sal*I sites of pGBT9.BS and pGAD.GH vectors [51], respectively. The complete coding region of SlCIPK24 cDNA was cloned into *BamH*I/*Sal*I sites of pGBT9.BS and pGAD.GH, resulting in pGBT⋅SlCIPK24 and pGAD⋅SlCIPK24, respectively. To create pGAD⋅SlCIPK24N and pGAD⋅SlCIPK24C deletion constructs, the corresponding SlCIPK24 regions were PCR amplified with the following primer sets, SlCIPK24-1/SlCIPK24-3 and SlCIPK24-4/SlCIPK24-2, digested with *BamH*I and *Sal*I restriction enzymes, and respectively cloned into the pGAD.GH vector.

For BiFC assays in onion epidermal cells, the SlCBL4 coding region lacking a stop codon was amplified with SlCBL4-5 and SlCBL4-6 primers and cloned into the *Sal*I/*Xho*I, sites of pSPYNE-35S vector, producing pSPYNE-35S⋅SlCBL4 (SlCBL4-YN) plasmid. The pSPYCE-35S⋅SlCIPK24 (SlCIPK24-YC) construct was generated by cloning the SlCIPK24 PCR product, amplified with SlCIPK24-6 and SlCIPK24-7 primers, into the *Xba*I/*Bam*HI sites of pSPYCE-35 vector [31]. The SlCBL4:c-Myc region in the pSPYNE-35S⋅SlCBL4 plasmid was amplified with SlCBL4-1 and c-Myc-2, digested with *EcoR*I and *Not*I, and cloned into the pGEX-4T-3 vector (GE Healthcare Life Sciences) to produce the pGEX⋅SlCBL4:c-Myc construct. In addition, the pGEX⋅SlCIPK24 construct was created by cloning the PCR product amplified with SlCIPK24-5 and SlCIPK24-2 primers into the *BamH*I/*Sal*I sites of pGEX-4T-3.

For creation of the SlCBL4-GFP fusion construct (pCAM⋅SlCBL4-GFP), the SlCBL4 coding region lacking a stop codon was amplified with a primer set of SlCBL4-3 and SlCBL4-4. The resulting PCR product was cloned into the *Nco*I/*Spe*I sites of the pCAMBIA1304 binary vector that contains a GFP reporter gene (CAMBIA, Australia). In a similar way, SlCIPK24-GFP (pCAM⋅SlCIPK24-GFP) was constructed with a primer set of SlCBL4-8 and SlCBL4-9. To make SlCBL4 overexpression construct (pATC⋅SlCBL4), the coding sequence of SlCBL4 cDNA was amplified with SlCBL4-7 and SlCBL4-8 primers, digested with *Spe*I and *Sac*I restriction enzymes, and cloned into the *Xba*I*/Sac*I sites of pATC940 vector under the control of strong constitutive promoter [61]. The pATC⋅SlCIPK24M construct which can overexpress a superactive SlCIPK24 mutant form in plants was created using pGAD⋅CIPK24T168D plasmid as template. The PCR product generated with SlCIPK24-6 and SlCIPK24-10 primers was cloned into the *Xba*I*/Not*I sites of pATC940 vector. All PCRs were carried out using *Pfu* DNA polymerase (Stratagene, La Jolla, CA, USA) and the constructs above were verified by DNA sequencing.

### 4.15. Oligonucleotide Primers

Primers used in this study for cloning and RT-qPCR are listed in Table S1 and Table S2, respectively.

## Data Availability

Not applicable.

## Supplementary Materials

The following are available online at https://www.mdpi.com/article/10.3390/plants10102173/s1, Figure S1: Isolation of transgenic tomato (cv. Moneymaker) plants overexpressing SlCBL4 or superactive SlCIPK24 mutant. Figure S2: Salt stress tolerance of the mature transgenic plants. Table S1: Primers used for plasmid construction. Table S2: Primers used for quantitative real-time RT-PCR.
