# Supplementary material for: Calcium Sensor SlCBL4 Associates with SlCIPK24 Protein Kinase and Mediates Salt Tolerance in Solanum lycopersicum
Source: Plants (Basel). 2021 Oct 14;10(10):2173. doi: 10.3390/plants10102173 (PMC8541381; doi:10.3390/plants10102173)
Supplement: Supplementary file 1 [file plants-10-02173-s001.zip › plants-1391665-supplementary.pdf]

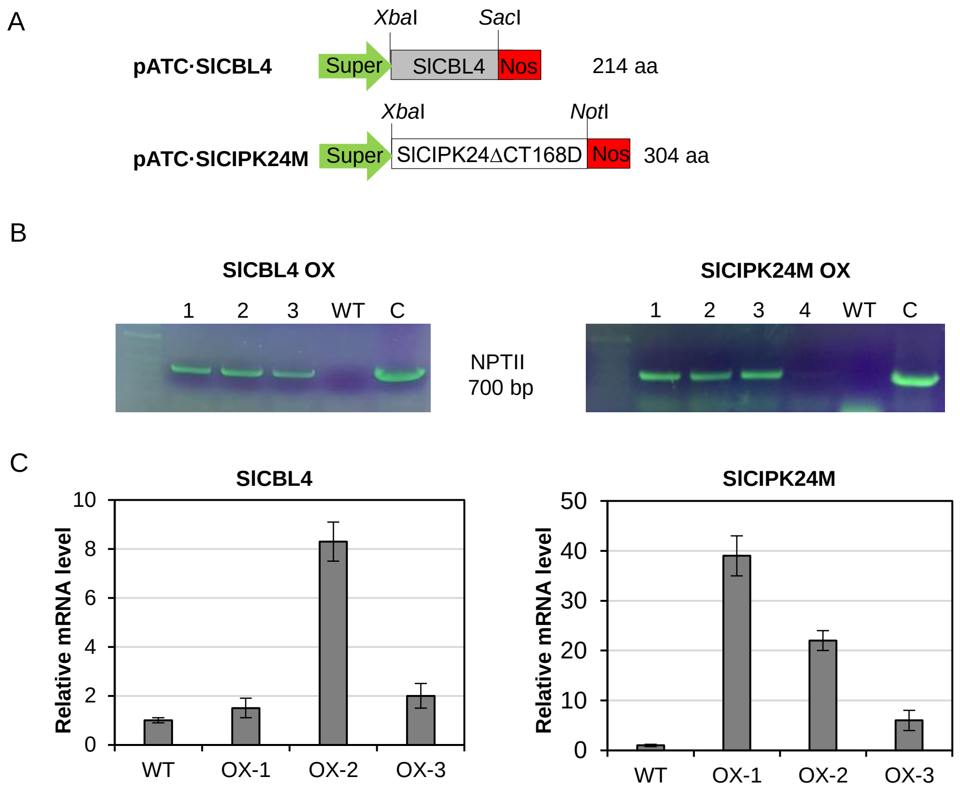


**Supplemental Figure S1.** Isolation of transgenic tomato (cv. Moneymaker) plants overexpressing SlCBL4 or superactive SlCIPK24 mutant. (A) Schematic diagrams of the pATC·SlCBL4 and pATC·SlCIPK24M. (B) PCR analysis showing the presence of the overexpression constructs in the transgenic lines. Genomic DNAs prepared from the indicated plants was amplified with a pair of primers (NPTII-F: 5'- GCTGCTCTGATGCCGCCGTGTTCCG-3', NPTII-R: 5'-AACTCGTCAAGAAGGCGATAGAAGGC -3) to produce a 700 bp fragment of the kanamycin resistance gene (NPTII). Lane C contains the PCR product from the plasmid template and serves as a positive control. (C) Real-time qRT-PCR analysis of the transcript levels in the WT and transgenic tomato lines. Total RNA was extracted from 3-week-old tomato plants. *SlActin7* was employed as an internal control for transcript normalization. The expression levels of *SlCBL4* and *SlCIPK24* in WT plants were respectively set to 1.0 to show their relative abundance in the transgenic plants. Error bars denote standard deviation (SD) of three technical replicates. Values are significantly different (the Student's *t*-test, $P \leq 0.05$).

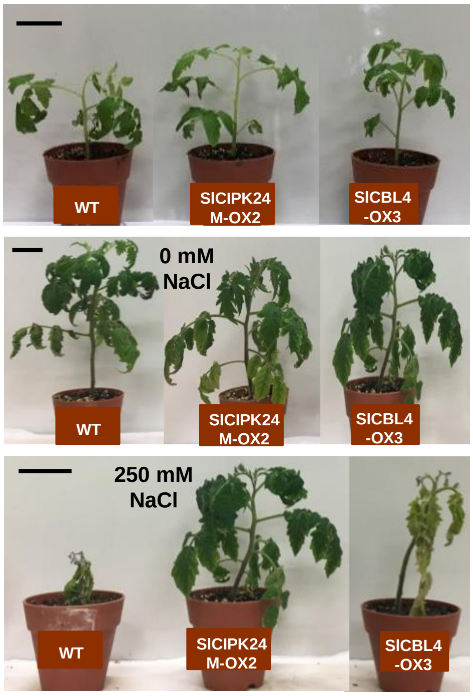


**Supplemental Figure S2. Salt stress tolerance of the mature transgenic plants.** Three-week-old plants (top panel) of the indicated lines were treated with 0 mM (middle panel) or 250 mM NaCl solution (bottom panel). The photos were taken on the 21st day after treatment. *Scale bars*, 5 cm. All the experiments were performed in three biological replicates.

**Supplemental Table S1.** Primers used for plasmid construction. Restriction enzyme sites were underlined. Three additional bases were included at the 5′ end of the primers for efficient restriction enzyme digestion.

| Primers | Sequences 5' to 3' (Restriction enzyme site) |
|---|---|
| SlCBL4-1 | TATGAATTCGATGGGCTGCTTTCCCTCAA (EcoRI) |
| SlCBL4-2 | TTTGTCGACCTAGACTTCCGAATCTTCCA (SalI) |
| SlCBL4-3 | AAACCATGGTAATGGGCTGCTTTCCCTCA (NcoI) |
| SlCBL4-4 | AAAACTAGTGACTTCCGAATCTTCCACCT (SpeI) |
| c-Myc-2 | AAAGCGGCCGCAAGATCCTCCTCAGAAATCA (NotI) |
| SlCBL4-5 | TATGTCGACATGGGCTGCTTTCCCTCAAA (SalI) |
| SlCBL4-6 | TTACTCGAGGACTTCCGAATCTTCCACCT (XhoI) |
| SlCBL4-7 | ATAACTAGTATGGGCTGCTTTCCCTCAAA (SpeI) |
| SlCBL4-8 | TAAGAGCTCCTAGACTTCCGAATCTTCCA (SacI) |
| SlCIPK24-1 | AAAGGATCCCATGAAGAAAGTGAAGAGAA (BamHI) |
| SlCIPK24-2 | AATGTCGACTCAGCGAGTCCTTGTCCTAA (SalI) |
| SlCIPK24-3 | TTTGTCGACTTACTCACTATCTTCTGATTTTT (SalI) |
| SlCIPK24-4 | TCTGGATCCGAGTGGTCCCTTGGTAATGA (BamHI) |
| SlCIPK24-5 | AAAGGATCCATGAAGAAAGTGAAGAGAAA (BamHI) |
| SlCIPK24-6 | AAATCTAGAATGAAGAAAGTGAAGAGAAA (XbaI) |
| SlCIPK24-7 | AATGGATCCGCGAGTCCTTGTCCTAAGCA (BamHI) |
| SlCIPK24-8 | AAACCATGGTAATGAAGAAAGTGAAGAGA (NcoI) |
| SlCIPK24-9 | AAAACTAGTGCGAGTCCTTGTCCTAAGCA (SpeI) |
| SlCIPK24-10 | TTTGCGGCCGCACTCACTATCTTCTGATT (NotI) |
| SlCIPK24-TD1 | GTCGAGCTCCTCTATGACACTTGTGGGACTCC |
| SlCIPK24-TD2 | GGAGTCCCACAAGTGTCATAGAGGAGCTCGAC |

**Supplemental Table S2.** Primers used for quantitative real-time RT-PCR.

| Primers | Sequence 5' to 3' | Size |
| --- | --- | --- |
| SlCBL4 Forward | CTGCTGGCTGCTGAGACCGCTTTTA | 181 bp |
| SlCBL4 Reverse | CAAATAGATCAAAAATCCTGTCTGC | |
| SlCIPK24 Forward | TAAGCATAGAATGGTTGAACAGATC | 179 bp |
| SlCIPK24 Reverse | CAGAAAGCCTACCTAGATGAACAAT | |
| SlRD29B Forward | GTCACCGATCCAACAGGCGCCAACA | 224 bp |
| SlRD29B Reverse | CCAACCGAGGGGAAAACAGTAGGCG | |
| SlRD22 Forward | GAGAATGTGAAGAGCCAGGTATTA | 191 bp |
| SlRD22 Reverse | GTCATTGTTGTTTCCCATTTTCTTG | |
| TAS14 Forward | TCGATCCGACAGCTCTAGCTCGTCG | 172 bp |
| TAS14 Reverse | AGGGATCTTGTCCTTGATTTTGTCC | |
| SlActin7 Forward | TGCTAGCGGTCGTACCACTGGTATT | 181 bp |
| SlActin7 Reverse | TACAATTTCCCGTTCAGCAGTAGTG | |
| SlTubulin Forward | AACCTCCATTCAGGAGATGTTTAGA | 180 bp |
| SlTubulin Forward | TCTGCTGTAGCATCCTGGTATTGAA | |
